# Differences in neurochemical profiles of two gadid species under ocean warming and acidification

**DOI:** 10.1186/s12983-017-0238-5

**Published:** 2017-10-30

**Authors:** Matthias Schmidt, Heidrun Sigrid Windisch, Kai-Uwe Ludwichowski, Sean Lando Levin Seegert, Hans-Otto Pörtner, Daniela Storch, Christian Bock

**Affiliations:** 1 Alfred-Wegener-Institute Helmholtz-Centre for Polar- and Marine Research, Section Integrative Ecophysiology, Am Handelshafen 12, 27570 Bremerhaven, Germany; 2 Department of Biology/Chemistry, University of Bremen, PO 330440, 28334 Bremen, Germany; 3 Institute for Cell Biology and Zoology, Heinrich Heine University, Universitätsstraße 1, 40225 Düsseldorf, Germany; 4 Alfred-Wegener-Institute Helmholtz-Centre for Polar- and Marine Research, Section Chemical Ecology, Am Handelshafen 12, 27570 Bremerhaven, Germany

**Keywords:** Ocean acidification, Temperature, ^1^H–NMR-spectroscopy, Untargeted metabolic profiling, HPLC, GABA

## Abstract

**Background:**

Exposure to future ocean acidification scenarios may alter the behaviour of marine teleosts through interference with neuroreceptor functioning. So far, most studies investigated effects of ocean acidification on the behaviour of fish, either isolated or in combination with environmental temperature. However, only few physiological studies on this issue were conducted despite the putative neurophysiological origin of the CO_2_-induced behavioural changes. Here, we present the metabolic consequences of long-term exposure to projected ocean acidification (396–548 μatm *P*CO_2_ under control and 915–1272 μatm under treatment conditions) and parallel warming in the brain of two related fish species, polar cod (*Boreogadus saida*, exposed to 0 °C, 3 °C, 6 °C and 8 °C) and Atlantic cod (*Gadus morhua*, exposed to 3 °C, 8 °C, 12 °C and 16 °C). It has been shown that *B. saida* is behaviourally vulnerable to future ocean acidification scenarios, while *G. morhua* demonstrates behavioural resilience.

**Results:**

We found that temperature alters brain osmolyte, amino acid, choline and neurotransmitter concentrations in both species indicating thermal responses particularly in osmoregulation and membrane structure. In *B. saida,* changes in amino acid and osmolyte metabolism at the highest temperature tested were also affected by CO_2_, possibly emphasizing energetic limitations. We did not observe changes in neurotransmitters, energy metabolites, membrane components or osmolytes that might serve as a compensatory mechanism against CO_2_ induced behavioural impairments. In contrast to *B. saida*, such temperature limitation was not detected in *G. morhua*; however, at 8 °C, CO_2_ induced an increase in the levels of metabolites of the glutamate/GABA-glutamine cycle potentially indicating greater GABAergic activity in *G.morhua*. Further, increased availability of energy-rich substrates was detected under these conditions.

**Conclusions:**

Our results indicate a change of GABAergic metabolism in the nervous system of *Gadus morhua* close to the optimum of the temperature range. Since a former study showed that juvenile *G. morhua* might be slightly more behaviourally resilient to CO_2_ at this respective temperature, we conclude that the observed change of GABAergic metabolism could be involved in counteracting OA induced behavioural changes. This may serve as a fitness advantage of this respective species compared to *B. saida* in a future warmer, more acidified polar ocean.

**Electronic supplementary material:**

The online version of this article (10.1186/s12983-017-0238-5) contains supplementary material, which is available to authorized users.

## Background

Exposure to projected CO_2_-induced ocean acidification (OA) scenarios alters the behaviour of some marine teleost species [1]. It has been suggested that these behavioural changes originate as a side effect of acid-base regulatory processes, which include extra- and intracellular bicarbonate accumulation associated with an equivalent reduction of chloride ions [2]. As a consequence, the electrochemical gradient of neurons in the central nervous system alters. This process is believed to affect functioning of γ-aminobutyric acid type A receptors (GABA_A_-R). Several experimental approaches support this hypothesis of altered GABA_A_-R activity [1, 3–5]. An altered functioning of the most important inhibitory neurotransmitter within the central nervous system, with great regulatory importance for neuronal circuits may lead to profound changes in neuronal activity and thus energetic demand. Acid-base regulatory processes are suggested to be responsible for altered fish behaviour, but this does not concern all species. Some species have been found to be more resilient to environmental CO_2_ than others, with unclear physiological background [6–9].

In this study, we assessed the question how chronic exposure to increased environmental CO_2_ affects metabolism in the brain of vulnerable *Boreogadus saida* and more resilient *Gadus morhua* [6, 8, 9]. Investigation of CO_2_ in combination with temperature effects should reveal future impacts of climate change in these ecologically and economically relevant species. Around Svalbard, the sea surface temperature is currently between −1.5 °C in winter and 8 °C in summer [10, 11], and is projected to rise by up to 2.5 °C until the year 2100 [12].The distribution of *G. morhua* currently shifts northward [13] and already overlaps with the distribution of *B. saida* in the seas around Svalbard with uncertain ecological consequences [10]. While direct commercial interest in *B. saida* is only minor, compared to that for *G. morhua*, its importance lies mainly in its function as forage for several other utilized fish species [14, 15]. Both, *B. saida* and *G. morhua*, will experience further warming and acidification until the end of the twenty-first century. Behavioural consequences of CO_2_ during concomitant warming have so far only been assessed in a few fish species [16–18]. Furthermore, studies of the combined effects of these two factors on brain metabolism have not been conducted at all.

**Fig. 1:**
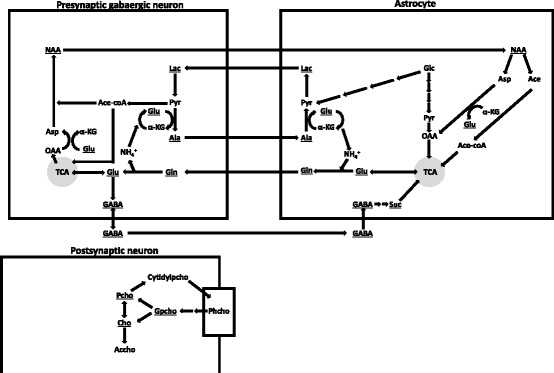
top: Metabolites involved in the GABA-glutamine cycle for neurotransmitter regeneration and the connected amino acid shuttle for ammonia transfer between presynaptic GABAergic neurons and surrounding astrocytes. Bottom: Metabolism of the membranous phosphatidylcholine as described by Klein [58]. Upon excitation, GABAergic neurons release GABA into the synaptic cleft, from where a minor fraction is taken up by the releasing GABAergic neuron itself and a major fraction by surrounding astrocytes. Within astrocytes it is catabolized to succinate which fuels the tricarbonic acid cycle. α-ketoglutarate of the TCA cycle is metabolized to glutamate and further aminated to glutamine, which is transported transcellularly into the GABAergic neuron. In the neuron, Glutamine is deaminated to glutamate and further decarboxylated to GABA which is again packed into synaptic vesicles. Lactate serves as neuronal energy source and is transported from astrocytes into neurons, where it is oxidized to pyruvate which subsequently enters the TCA cycle after transformation into acetyl-coA. A fraction of cellular pyruvate is aminated to alanine which is transported back to astrocytes in order to avoid accumulation of ammonia inside the neuron. N-acetylaspartate, which is generated in neurons from aspartate and acetyl-coA, can re-enter the TCA cycle of astrocytes as oxaloacetate under energy-deprived conditions. Phosphatidylcholine is present in all cell membranes of neurons and astrocytes but for the sake of clarity, its metabolism is displayed in the postsynaptic neuron only. As described by Bak et al. [35] membranous phosphatidylcholine gets catabolized to dissolved choline or alternatively, phosphocholine via glycerophosphocholine. In cholinergic neurons choline can be utilized for anabolism of the neurotransmitter acetylcholine, while phosphocholine can be used for regeneration of phosphatidylcholine via cytidylphosphocholine. Underlined metabolites were quantified through ^1^H–NMR spectroscopy. The scheme is adapted after Bak et al. [35] and Klein [58]. Ace = Acetate; Ace-coA = Acetyl-coA; Accho = Acetylcholine; α-KG = Alpha ketoglutaric acid; Ala = Alanine; Asp = Aspartate; Cho = Choline; Cytidylpcho = Cytidylphosphocholine; GABA = Gamma-aminobutyric acid; Glc = Glucose; Gln = Glutamine; Glu = Glutamate; Gpcho = Glycerophosphocholine; Lac = Lactate; NAA = N-acetylaspartate; OAA = Oxaloacetic acid; Pcho = Phosphocholine; Phcho = Phosphatidylcholine; Pyr = Pyruvate; Suc = Succinate; TCA = Tricarbonic acid cycle

The present study focuses particularly on metabolites and amino acids involved in energy metabolism and regeneration of the neurotransmitter γ-aminobutyric acid (GABA) in order to test whether compensatory mechanisms to a rise of environmental CO_2_ are visible on neurotransmitter level. In addition, we studied the neurotransmitter serotonin (5-HT) and its catabolite since serotonergic activity is positively correlated with chronic stress in fish [19]. Compounds involved in the metabolism of phospholipids were also analysed since Leo et al. found that CO_2_ might affect proton leakage in mitochondria of *G. morhua* and other teleosts [20, 21]. The networks of tested metabolites involved in GABA and phospholipid metabolism are displayed in Fig. 1. As exposure to increased CO_2_ also alters the composition of dissolved extra- and intracellular ion species in the brain of fish [5] we also took osmolyte concentrations into account to fully track changes in brain metabolites under elevated CO_2_ and at various temperatures.

## Methods

### Specimen collection and maintenance

Juvenile *Boreogadus saida* were caught on January 17th 2013 in the inner Kongsfjord (78.97 N, 12.51 E) at an approximate depth of 120 m. They were housed in aquaria of the Tromsø Aquaculture Research Station, in Kårvik, Norway before being transported to the Alfred Wegener Institute in Bremerhaven, Germany (AWI). Juvenile *Gadus morhua* were collected between the 26th and 29th of August 2013 during a cruise of the RV Heincke around Hinlopenstretet (79.30 N, 18.57 E), Rijpfjorden (80.15 N, 22.12 E), and Forlandsundet (78.54 N, 11.3 E) and were subsequently transported to the AWI. Additional information on the cruise is provided under http://doi.pangaea.de/10.1594/PANGAEA.824703. Animals were kept in a recirculating seawater system at the AWI at 5 °C and environmental *P*CO_2_ until onset of the incubations.

### Incubations

Extensive information on the incubation and animals has been provided by Kunz et al. [22]. Incubation periods were 102–114 days for *B. saida* and 111–132 days for *G. morhua*. *B. saida* (length: 14.4 ± 1.1 cm; weight: 18.0 ± 4.9 g) was incubated at 0 °C, 3 °C, 6 °C and 8 °C and *G. morhua* (length: 18.0 ± 2.0 cm; weight: 39.5 ± 14.9 g) at 3 °C, 8 °C, 12 °C and 16 °C. Temperatures were chosen in order to represent a wide range of the thermal habitat. At each temperature, animals were kept either at ambient control *P*CO_2_ (396–548 μatm.) or high *P*CO_2_ (915–1272 μatm.), as projected for the year 2100 [12]. The factorial design was comprised of 8 different treatment groups per species. Means and standard deviations of *P*CO_2_ for each treatment group are presented in Additional file 1: Table S1. Temperatures were adjusted by a maximum of 2 °C per day beginning at 5 °C. Afterwards, CO_2_ conditions were adjusted during a single day. The incubation started after the desired temperature and CO_2_ conditions had been reached. Animals were kept in individual tanks to avoid cannibalism and changes in the neurochemical profile due to dominance hierarchies.

A gas mixing system (HTK, Germany) was used for adjustment of *P*CO_2_. Temperature, salinity, dissolved inorganic carbon (DIC) and pH_tot_ were measured at least once per week. Extensive information on the methodology of carbonate chemistry measurements and raw data are available under https://doi.pangaea.de/10.1594/PANGAEA.866369. A summary of the carbonate chemistry for the whole incubation period has been published by Kunz et al. [22].

### Tissue sampling and preparation

After incubation, animals were exposed to surgical anaesthesia at 200 mg/l MS-222 in a water bath containing seawater from aquaria of the respective treatment group. When the fish did not respond anymore to external stimuli, they were taken out of the water bath and sacrificed though cervical dislocation. Brains were removed, transferred to centrifuge tubes, rapidly frozen in liquid nitrogen and subsequently stored at −80 °C. In order to enable NMR and HPLC analysis on the same tissue samples, each brain was powdered with mortar and pestle under liquid nitrogen. The grind was well mixed and aliquots of ~50 mg were taken for NMR and HPLC analysis. Brains of three to six animals per treatment group were separately analysed via NMR and HPLC using one aliquot for NMR and one aliquot for HPLC measurements. The remaining brains were utilized in experiments not covered by the scope of this paper.

### Untargeted metabolic profiling (using ^1^H–NMR-spectroscopy)

Extraction of brain tissue was conducted after Belle et al. [23]. Briefly, one aliquot of powdered brain tissue was transferred into a cooled glass centrifuge tube on ice. 3 ml ice-cold Dichloromethane/Methanol (2:1 *v*/v) were added quickly and the suspension mixed. The tube was sonicated for ten minutes at 4 °C in a Branson sonifier with 50% duty cycle. Subsequently, 1 ml 0.88% KCl (*w*/w) was added, the suspension was mixed again and centrifuged for ten minutes at 805 g. The upper methanol phase was transferred to another centrifuge tube and the solution dried overnight in a vacuum concentrator (RVC 2–18 HCl, Christ GmbH Osterode, Germany). The remaining pellet was stored for two to three days at 4 °C in a fridge before being re-suspended in D_2_O (Deuterium oxide) containing 0.05% TSP (Trimethylsilylpropionate) as internal standard. Two to four fold the weight of the original grind of D_2_O was added in a volume equivalent (depending on the amount of initial brain tissue) to suspend the pellet. Untargeted metabolic profiling based on ^1^H–NMR spectroscopy was performed on a wide-bore 400 MHz NMR spectrometer (9.4 T WB with Avance III HD electronics, Bruker Biospin, Germany) using a triple tuned ^1^H-^13^C-^31^P-HRMAS NMR probe. A sample volume of ~ 50 μl was filled in a standard zirconium rotor for high-resolution magic angle spinning (HRMAS) NMR spectroscopy. All NMR spectra were conducted at a spinning rate of 3000 Hz and a sample temperature of 10 °C. Four different ^1^H–NMR measurements were collected for all samples, consisting of a standard one pulse 1D ^1^H NMR spectroscopy with f1 pre-saturation, a 1D Carr-Purcell-Meiboom-Gill (CPMG) pulse train including f1 pre-saturation (Bruker protocol cpmgpr1d), a NOESY sequence and a pseudo 2D ^1^H-^1^H J-resolved (JRES) NMR spectroscopy protocol for metabolite identification. All metabolite profiles were analysed from the CPMG NMR protocol with the following acquisition parameters: pulse length 8.4 μs for 90°, time domain 70,656, sweep width of 8802 Hz (22 ppm), acquisition time 4.01 s, relaxation delay 4 s, four dummy scans and 64–256 number of scans depending on the signal-to-noise ratio.

Samples were analysed in randomized order to avoid systematic errors. All spectra were analysed using Chenomx NMR suite 8.1 (Chenomx Inc., Canada). Data were automatically zero filled to at least 128 k and processed with an exponential multiplication of 0.3 Hz. After phase and baseline correction line-shape distortions were eliminated through shim correction. Metabolites were identified using the Chenomx data base and an online spectral data base for organic compounds (http://sdbs.db.aist.go.jp, SDBS, National Institute of Advanced Industrial Science and Technology (AIST)). NMR peak integrals were fitted manually to the specific metabolites for quantification. In total, 24 compounds were identified: Acetate, creatine, lactate, phosphocreatine and succinate are mainly part of cellular energy metabolism; alanine, GABA, glutamine and glutamate are involved in metabolism of GABA either as part of the GABA-glutamine cycle or the lactate-alanine shuttle (lactate is also listed among the metabolites involved in energy metabolism). Compounds involved in the metabolism of phosphatidylcholine are choline, glycerophosphocholine, phosphocholine and putrescine. Acetyl-histidine, myo-inositol (MI), N-acetylaspartate (NAA), taurine and trimethylamine-*N*-oxide (TMAO) serve as osmolytes in the central nervous system. In addition to its function as osmolyte, NAA is further involved in energy metabolism (Fig. 1). Glycine was quantified as it serves together with GABA as important inhibitory neurotransmitter throughout the central nervous system [24]. Peaks of ascorbate, aspartate, threonine and valine were mostly visible, however, either close to detection limit, or strongly overlapping with other, more prominent, compounds. As a reliable quantification of these substances was not possible, they were excluded from subsequent analysis. Methanol is a trace compound from the extraction process and was therefore also not further analysed. The spectrum of acetyl-histidine was not available in the Chenomx data base and was created manually in the Chenomx compound builder prior to analysis. We detected strong differences in metabolite concentrations between samples, which are likely caused by different extraction efficiencies. To compensate for this bias, we calculated for each spectrum total creatine (tCr) - the sum of creatine and phosphocreatine- as internal reference. Afterwards, the concentration of each metabolite relative to the concentration of tCr was calculated and these ratios were used for statistical analysis. For *Boreogadus saida*, the brains of 38 individuals were analysed, for *Gadus morhua*, the brains of 40 individuals. The exact group sizes are document in Additional file 1: Table S1.

### HPLC-analysis

Deionized water was utilized for preparation of all buffers. All chemicals used were either of HPLC-grade of the highest purity available. HPLC-analysis of brain tissue was conducted with a modified method of Yoshitake et al. [25] through derivatization with benzylamine (BA) and diphenylethylenediamine (DPE) followed by subsequent fluorescence detection. BA was dissolved 0.3 M in an aqueous solution with 90% methanol (*v*/v). A 0.3 M CAPS buffer was prepared in aqueous 90% methanol (*v*/v) and subsequently adjusted to a pH of 11 with 10 M NaOH. Potassium hexacyanoferrate (III) was prepared in an aqueous 50% methanol (*v*/v). A 0.2 M DPE solution was prepared in methanol and subsequently diluted by 50% with 0.2 M aqueous HCl. Glycine was prepared 0.3 M in H_2_O. Two derivatisation reagents were made: First, derivatisation reagent “A” containing the above prepared BA, CAPS, potassium hexacyanoferrate solutions and methanol in a stoichiometry of 2:6:3:24 (v/v/v/v). Second, derivatisation reagent “B” containing the prepared DPE and glycine solutions in a stoichiometry of 2:1 (v/v).

Powdered brain tissue in a centrifuge cup was extracted by adding 0.1 N ice-cold aqueous perchloric acid with 10^−7^ M ascorbic acid at a volume of ten-fold the weight of the grind. The cup was mixed and sonicated for two minutes at 4 °C in a Branson sonifier with 50% duty cycle. The cup was subsequently centrifuged at 5200 g for 30 min at 4 °C and the supernatant transferred to a different centrifuge cup. The supernatant was brought to a pH of ~4.5 through addition of 0.5 M ice-cold KOH and subsequently centrifuged again for 30 min at 4 °C.

Reversed phase solid phase extraction was conducted with Oasis HLB cartridges (Waters Corporation, Milford, USA), containing 30 mg sorbent per cartridge with 1 ml methanol as solvent for the eluate. The eluate was dried overnight in a centrifuge cup in a vacuum concentrator (RVC 2–18 HCl, Christ GmbH Osterode, Germany). On the next day, the pellet was dissolved in 200 μl H_2_O. 200 μl of derivatisation reagent “A” was added and allowed to react for two minutes at room temperature. Subsequently, 200 μl of derivatisation reagent “B” was added and the mixture incubated for 20 min at 50 °C after which it was rapidly cooled on ice. The solution was filtered through a 20 μm filter and transferred into an amber-coloured glass vial. Measurements were conducted overnight in a HPLC system at room temperature with a LaChrom Elite® (Hitachi High Technologies America, USA) on a reversed phase Kinetex C18 column (Phenomenex, length: 150 mm; diameter: 4.6 mm; particle size: 2.6 μm). We achieved separation through a non-isocratic elution with fractions of two media: first, a 15 mM sodium acetate, 1 mM octanesulfonic acid buffer (pH 4.5) that was diluted with acetonitrile (1:2 (*v*/v)) and, second, pure acetonitrile. The used proportions are available in Additional file 2: Table S2. Measurement time was 90 min per sample at a flow rate of 0.6 ml/min. Components were identified through their specific retention times using a fluorescence detector with an excitation wavelength of 345 nm and emission wavelength of 480 nm. Using this method, we were able to quantify norepinephrine, 5-hydroxytryptophan, 5-hydroxyindoleacetic acid (HIAA), serotonin (5-hydroxytryptamine (5-HT)), dopamine, 3,4-dihydroxyphenylacetic acid (DOPAC) and L-3,4-dihydroxyphenylalanine (L-DOPA) in standard solutions. However, in tissue samples, only 5-HT, HIAA and DOPAC were reliably detectable. The other substances were either only visible in very small amounts or not at all. Addition of the respective substances to brain tissue prior to solid phase extraction did not enable their detection suggesting a methodological issue rather than low concentration in the brain tissue. We thus focused our analysis on the serotonergic pathway containing serotonin and its catabolite HIAA. For *Boreogadus saida* and *Gadus morhua*, the brains of 39 individuals per species were analyzed. The exact group sizes are document in Additional file 1: Table S1.

### Statistical analysis

Statistical analysis was conducted with “R” (v. 3.2.3). Log-transformed metabolite/tCr ratios from NMR analysis and log-transformed HIAA/5-HT ratios from HPLC analysis of each species were tested separately for temperature-, CO_2_- and their interactive effects using ordinate two-way-ANOVAs (α = 0.05). Tukey HSD (α = 0.05) from the package “agricolae” (v. 1.2–3) was used for post hoc multiple comparison testing. Normality distribution of metabolite/tCr and HIAA/5-HT ratios were evaluated for each treatment group using Shapiro Wilk normality test (α = 0.05), variance homogeneity with a Bartlett test (α = 0.05). Non-metric multidimensional scaling with stable solution from random starts (“metaMDS”) was conducted on metabolite/tCr ratios with the package “vegan” (v. 2.3–3) in order to test for overall temperature- and CO_2_-effects among individuals. The stress level of the metaMDS was in an acceptable range (~0.1). Violation of normality distribution was observed in 17 out of 272 groups tested. This may be due to the sheer amount of observations tested with an α of 0.05. The cumulative random chance of observing 17 or more normality violations under this condition is ~14% on the basis of a binomial distribution, which is thus not significantly different from what could be expected by chance. Violation of normality may still yield implications for possible type I and II errors in the applied ANOVAs; however, we did not see justification for removal of the outlying data points, especially in consideration of the small sample size. A list of the groups with violated normality distribution is provided in Additional file 3: Table S3. Variance homogeneity was violated on three occasions: Glycine/tCr and lactate/tCr ratios of *B. saida* and the TMAO/tCr ratio of *G. morhua*.

## Results

Table 1 depicts a synopsis of temperature-, CO_2_- and interactive effects on the analysed components. Boxplots of those components influenced either by CO_2_ or interactively by CO_2_ in combination with temperature are shown in Fig. 3 (*Boreogadus saida*) and Fig. 4 (*Gadus morhua*). Complementary boxplots of all compounds, including those affected by temperature only, are available in the Additional file 4: Figure S1 and Additional file 5: Figure S2 for NMR-data, Additional file 6: Figure S3 for HPLC-data.

**Table 1:** Summary of temperature and CO_2_-related effects and their interaction on compounds analysed via ^1^H–NMR-Spectroscopy and HPLC

		*Boreogadus saida*			*Gadus morhua*		
Compound	Class	Temperature	CO_2_	Interaction	Temperature	CO_2_	Interaction
Ace	Energy metabolism	n.s.	n.s.	n.s.	n.s.	n.s.	n.s.
Lac	Energy metabolism	**,↓	n.s.	n.s.	n.s.	*↑	*
Suc	Energy metabolism	n.s.	n.s.	n.s.	n.s.	n.s.	n.s.
Ala	GABA metabolism	***,↓	n.s.	n.s.	***,↓	n.s.	n.s.
GABA	GABA metabolism	***,↓	*,↑	n.s.	***,↓	n.s.	*
Gln	GABA metabolism	*,↓	n.s.	n.s.	*,↑↓	n.s.	n.s.
Glu	GABA metabolism	***,↓	n.s.	***	***,↓	n.s.	n.s.
Cho	Membrane component	n.s.	n.s.	n.s.	n.s.	n.s.	**
Gpcho	Membrane component	***,↓	n.s.	n.s.	***,↓	n.s.	n.s.
Pcho	Membrane component	**,↑↓	n.s.	n.s.	***,↓	n.s.	n.s.
Put	Membrane component	***,↑↓	n.s.	n.s.	***,↓	n.s.	n.s.
AcHis	Osmolyte	n.s.	n.s.	n.s.	n.s.	n.s.	n.s.
MI	Osmolyte	***,↓	***,↑	n.s.	***,↓	n.s.	n.s.
NAA	Osmolyte	***,↑	n.s.	n.s.	***,↑	n.s.	n.s.
Tau	Osmolyte	**,↑↓	n.s.	n.s.	***,↓	n.s.	n.s.
TMAO	Osmolyte	***,↓	n.s.	n.s.	***,↓	n.s.	n.s.
Gly	Other	*,↓	n.s.	n.s.	***,↓	n.s.	n.s.
HIAA/5-HT	Other	*,↑	n.s.	n.s.	*,↑	n.s.	n.s.

Compound classes are assigned to match the grouping of each compound as used throughout the discussion of the manuscript

* = *p* < 0.05, ** = *p* < 0.01, *** = *p* < 0.001. **↑** and **↓** indicate either an increase or a decrease of the respective compound with rising temperature or rising CO_2_. **↑↓** indicates apparent uneven effects. Interactive effects are per definition uneven and were therefore not characterized. A plot for each component is available in the (Additional file 4: Figure S1 and Additional file 5: Figure S2 (NMR) and Additional file 6: Figure S3 (HPLC))

*Ace* Acetate, *AcHis* Acetyl-histidine, *Ala* Alanine, *Cho* Choline, *GABA* Gamma-aminobutyric acid, *Glu* Glutamate, *Gln* Glutamine, *Gpcho* Glycerophosphocholine, *Gly* Glycine, *Lac* Lactate, *MI* Myo-inositol, *NAA* N-acetylaspartate, *Pcho* Phosphocholine, *Put* Putrescine, *Suc* Succinate, *Tau* Taurine, *HIAA* 5-Hydroxyindoleacetic acid, *5-HT* 5-Hydroxytryptamine (Serotonine)

### *Boreogadus saida* - NMR

**Fig. 2:**
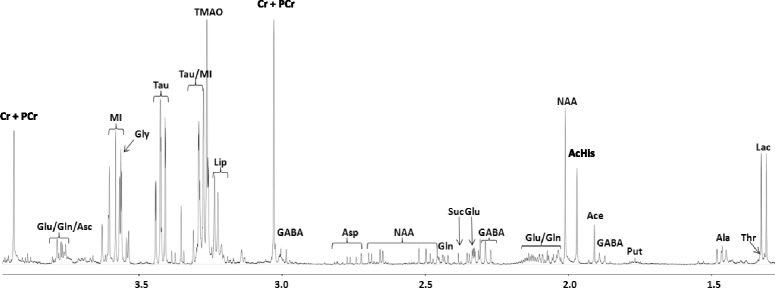
Exemplary ^1^H–NMR spectrum of a brain methanol/dichloromethane extract of *Boreogadus saida.* The x-axis represents the chemical shift of the respective compounds in parts per million (ppm). Ace = Acetate; AcHis = Acetyl-histidine; Ala = Alanine; Asc = Ascorbate; Asp = Aspartate; Cr + PCr = Creatine and Phosphocreatine; GABA = Gamma-aminobutyric acid; Gln = Glutamine; Glu = Glutamate; Gly = Glycine; Lac = Lactate; Lip = Lipids including choline, glycerophosphocholine and phosphocholine; MI = Myo-inositol; NAA = N-acetylaspartate; Put = Putrescine; Suc = Succinate; Tau = Taurine; Thr = Threonine; TMAO = Trimethylamine-N-Oxide

**Fig. 3:**
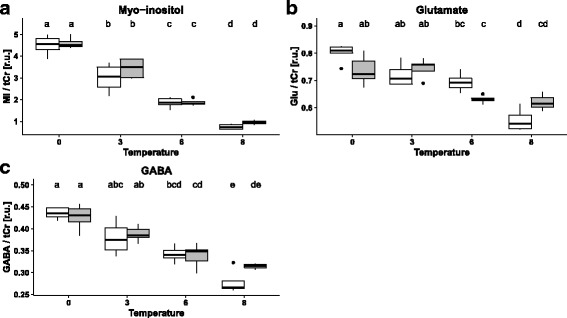
Boxplots depicting concentrations of metabolites affected by CO_2_ (direct or interactive effects, relative to total creatine (tCr), relative units [r.u.]) in the brain of *Boreogadus saida* at different temperatures and CO_2_ partial pressures. White shading indicates control, grey shading high CO_2_ partial pressure. Each box contains median, first and third quartile as well as respective standard deviation. Different letters (**a**, **b**, **c**, **d**, **e**) indicate significant differences between treatment groups detected by Tukey HSD post hoc analysis (*p* < 0.05)

Figure 2 presents a typical ^1^H–NMR cpmg spectrum from a methanol/dichloromethane extract of *Boreogadus saida* brain tissue. Temperature strongly affected most compounds tested in this species. Most evident changes were observable among osmolytes, in particular MI, the concentration of which was around 80% lower at 8 °C than at 0 °C (*p* < 0.001, F_3,30_ = 267.3, Fig. 3a). A similar reduction with increasing temperature was observed for TMAO (*p* < 0.001, F_3,30_ = 56.44). In a striking contrast, NAA increased with rising temperature by about 30% between 0 °C and 8 °C (*p* < 0.001, F_3,30_ = 39.06). Taurine exhibited the highest concentration at 6 °C, decreasing above and below this temperature (*p* < 0.01, F_3_,_30_ = 5.299). Acetyl-histidine was not influenced by temperature (*p* > 0.05). All tested substances directly involved in GABA metabolism showed a reduced concentration with increased temperature. This effect was most prevalent for glutamate and GABA (*p* < 0.001, F_3,30_ = 42.87 and *p* < 0.001, F_3,30_ = 52.37, Fig. 3b and c respectively). The effect of temperature on alanine and glutamine was weaker and mainly caused by a decrease from 6 °C to 8 °C (*p* < 0.001, F_3,30_ = 14.15 and *p* < 0.05, F_3,30_ = 4.417).

Among compounds representing energy metabolism only lactate was reduced through temperature increase (*p* < 0.01, F_3,30_ = 4.547), an effect which was particularly prevalent between 6 °C and 8 °C. Acetate and succinate were not affected by temperature (p > 0.05). Most membrane components tested were affected by temperature with the exception of choline (p > 0.05). Putrescine and phosphocholine concentrations followed a bell-shaped curve with highest concentrations at 3 °C and 6 °C (*p* < 0.001, F_3,30_ = 7.892 and *p* < 0.01, F_3,30_ = 6.693). Glycerophosphocholine concentrations decreased significantly with increasing temperature (*p* < 0.001, F_3,30_ = 87.42). Glycine was significantly reduced during warming (*p* < 0.05, F_3,30_ = 3.637) with a change particularly strong between 6 °C and 8 °C. Simultaneously, glycine displayed a reduction of variance with increasing temperature which was detected with Bartlett test for variance homogeneity.

CO_2_-effects were visible for the osmolyte MI and for GABA (*p* < 0.001, F_1,30_ = 23.58 and *p* < 0.05, F_1,30_ = 4.478). CO_2_ caused an increase in the concentrations of these compounds, mainly at 8 °C. However, CO_2_-effects were much lower than the observed temperature-related changes and post-hoc-tests therefore negative.

Interactive effects of temperature and CO_2_ were detected in the changes of glutamate content which is involved in GABA metabolism (*p* < 0.001, F_3,30_ = 7.217).

Non-metric multidimensional scaling supports the conclusion that the variance in metabolite levels is largely explainable through temperature effects (*p* < 0.001, R^2^ ~ 0.93, 10^3^ permutations, Additional file 7: Figure S4) with only a small contribution by CO_2_ (p ~ 0.6, R^2^ ~ 0.03, 10^3^ permutations).

### *Boreogadus saida* - HPLC

Warming caused a significant change in serotonin metabolism with an increase of the HIAA/5-HT ratio (*p* < 0.05, F_3,31_ = 3.525, Additional file 6: Figure S3A). Neither CO_2_, nor interactive effects with temperature were detected.

### *Gadus morhua* – NMR

**Fig. 4:**
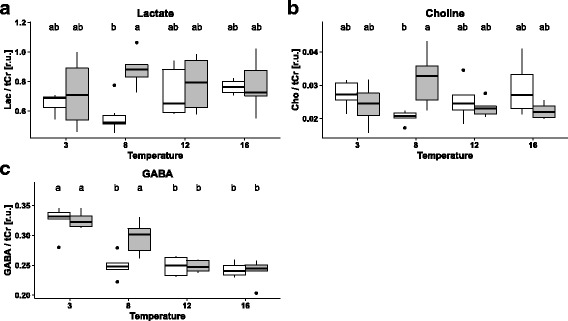
Boxplots depicting concentrations of metabolites affected by CO_2_ (direct or interactive effects, relative to total creatine (tCr)) in the brain of *Gadus morhua* at different temperatures and CO_2_ partial pressures. White shading indicates control, grey shading high CO_2_ partial pressure. Each box contains median, first and third quartile as well as respective standard deviation. Different letters (**a**, **b**) indicate significant differences between treatment groups detected by Tukey HSD post hoc analysis (*p* < 0.05)

As in *B. saida*, most compounds tested were affected by environmental temperature. The strongest changes were again observed among osmolytes. In particular, MI fell by >90% during warming from 3 °C to 16 °C (*p* < 0.001, F_3,32_ = 986.5). The greatest reduction by ~75% occurred between 3 °C and 8 °C. A similar reduction by ~90% between 3 °C and 16 °C was observed for TMAO with the greatest drop again between 3 °C and 8 °C (*p* < 0.001, F_3,32_ = 60.87). In contrast to MI and TMAO, NAA increased significantly by about 50% between 3 °C and 16 °C (*p* < 0.001, F_3,32_ = 70.95). Taurine concentrations were highest at 3 °C and 8 °C and dropped beyond 8 °C (*p* < 0.001, F_3,32_ = 12.97). Acetyl-histidine was not affected by temperature (*p* > 0.05). Similar to *B. saida*, concentrations of most compounds involved in GABA metabolism strongly decreased with increasing temperature. This temperature effect was again strongest for glutamate (*p* < 0.001, F_3,32_ = 87.38), followed in magnitude by GABA (*p* < 0.001, F_3,32_ = 32.16, Fig. 4c). A warming-induced reduction of alanine levels occurred mainly between 3 °C and 8 °C, as observed in *B. saida* (*p* < 0.001, F_3,32_ = 10.75). The temperature effect on glutamine was less clear. Means of glutamine at 12 °C and 16 °C were higher than at 3 °C and 8 °C, however, with the exception of the 3 °C control-CO_2_ group. Differences in glutamine levels between groups were rather small and post hoc tests negative (*p* > 0.05). The overall temperature effect though, was still slightly significant (*p* < 0.05, F_3,32_ = 4.370). Acetate, lactate and succinate levels were not altered by temperature (p > 0.05). As in *B. saida*, most components involved in membrane metabolism were affected by temperature, except for choline (p > 0.05, Fig. 4b). Putrescine, phosphocholine and glycerophosphocholine level fell during warming (*p* < 0.001, F_3,32_ = 11.28, *p* < 0.001, F_3,32_ = 78.03 and *p* < 0.001, F_3,32_ = 121.0). As in *B. saida*, glycine concentrations decreased with rising temperatures (*p* < 0.001, F_3,32_ = 40.17).

A significant CO_2_ effect was observed for lactate with an increase under high CO_2_ (*p* < 0.05, F_1,32_ = 7.24 Fig. 4a). This finding was mainly governed by a CO_2_-related increase of lactate at 8 °C (*p* < 0.05 in post hoc analysis) which led to detection of a significant interactive effect of temperature and CO_2_ (*p* < 0.05, F_3,32_ = 2.94). Interactive effects of temperature and CO_2_ were also detected for choline and GABA (*p* < 0.05, F_3,32_ = 5.870 and *p* < 0.05, F_3,32_ = 4.033 Fig. 4b and c). For these substances post hoc tests revealed a significant CO_2_-dependent increase at 8 °C (*p* < 0.05), which was absent at other temperatures.

As in *B. saida*, non-metric multidimensional scaling revealed for *G. morhua* that the vast majority of overall variance was explainable through temperature effects (*p* < 0.001, R^2^ ~ 0.95, 10^3^ permutations, Additional file 8: Figure S5), with only a minor contribution by CO_2_ (p ~ 0.16, R^2^ ~ 0.09, 10^3^ permutations).

### *Gadus morhua* - HPLC

As in *B. saida*, temperature significantly affected serotonin metabolism of *G. morhua* leading to a rise of the HIAA/5-HT ratio during warming (*p* < 0.05, F_3,31_ = 3.394, Additional file 6: Figure S3B). Neither were effects of CO_2_, nor interactive effects detected.

## Discussion

### Interference with anaesthesia

It cannot be fully excluded that additional factors such as the anaesthetic procedure may have an interactive effect with temperature or CO_2_ contributing to the here presented observations. Effects of MS-222 on the respiratory and cardiovascular system of fish are well documented as well as the rapid MS-222 induced release of cortisol, changing inter alia the ionic composition of blood plasma [26, 27]. Consequences of MS-222 for intracellular osmolytes and neurotransmitter systems have not been reported and would demand further investigation.

### Effect of temperature on brain metabolites

Temperature affected metabolite concentrations in the brain of *Boreogadus saida* and *Gadus morhua* in a very similar manner. The strongest alterations were visible among the osmolytes TMAO and MI, which fell in both species upon warming. This finding is in line with the view that TMAO and MI are cytoprotective in cold environments [28, 29]. In both species TMAO/tCr ratios reacted similarly to warming and underwent a reduction by ~ 70% from 3 °C to 8 °C. A striking difference was visible in the relative amount of TMAO, as at 3 °C and 8 °C the TMAO/tCr ratio in *B. saida* was around six-fold greater than the TMAO/tCr ratio of *G. morhua*, which indicates a greater importance of TMAO in physiological cold adaptation of *B. saida*. While temperature altered taurine concentrations in *B. saida* in a uneven manner*,* taurine decreased with increasing temperature in *G. morhua*. Osmolytes play a role in cytoprotection, and in both species elevated taurine concentrations may be beneficial below 8 °C [30] and may serve as compensation to the constitutive reduction of TMAO at temperatures above 0 °C. A decrease of osmolytes with increasing temperature was expected as high osmolarity in cold-adapted fish serves freezing point reduction [31]. In contrast, NAA levels increased in both species with increasing temperatures. In addition to its putative functions as osmolyte and potential energy reserve in neurons and oligodendrocytes, concentration changes of this substance might also have implications for membrane composition, as discussed below. However, not all osmotically active substances responded to temperature. Unlike Baslow and Guilfoyle [32] we did not detect temperature-related changes of acetyl-histidine concentrations as observed in killifish (*Fundulus heteroclitus*) and goldfish (*Carassius auratus*). However, the temperature range investigated in this study (0 °C - 16 °C) was narrower than the range analysed by Baslow and Guilfoyle (13.3 °C - 30 °C).

In addition to their function as synaptic neurotransmitters, GABA, glutamate and glycine influence osmotic relations [33] which may explain why their concentrations decreased continuously with rising temperatures as observed in the prominent osmolytes as discussed above. This may explain the temperature dependence of glutamate and GABA in both species, and of glycine in *G. morhua*. Their concentrations decreased continuously with rising temperature. Windisch et al. found a strong temperature dependency in the expression of the glycine cleavage system in Antarctic eelpout, which was much higher expressed at cold temperatures below 0 °C indicating importance of glycine metabolism at low temperatures [34]. Whether the glycine cleavage system serves at low temperatures the catabolism of excessive glycine or rather its anabolism demands further experimentation. In *B. saida*, reductions of lactate, alanine, and glutamine levels occurred mainly between 6 °C and 8 °C, potentially indicating energy limitations at 8 °C as not only lactate, but also glutamate, glutamine, GABA are energy sources for the tricarbonic acid (TCA) cycle in brain [35]. This hypothesis is supported by the fact that mortality of *B. saida* only occurred at 8 °C and that this temperature was recently identified to represent the long-term upper thermal tolerance limit for this species [22]. In *B. saida*, glycine concentrations were marginally reduced by increasing temperature, but the variance of concentrations between treatments fell with rising temperature, especially at 8 °C, potentially indicating energy limitations as well. A reduction of alanine levels at 8 °C in *B. saida* may not necessarily be a proxy for energy status, as *G. morhua* also exhibited a reduction of alanine level between 3 °C and 8 °C, but no further alteration of alanine concentrations was observed between 8 °C and 16 °C rather suggesting a temperature-dependent functioning of this amino acid as osmolyte.

Alterations of phosphocholine, glycerophosphocholine and putrescine with temperature may indicate structural changes in membrane metabolism and composition. Phosphocholine and glycerophosphocholine are intermediates of phosphatidylcholine metabolism and putrescine acts as progenitor for other polyamines such as spermidine and spermine, all of which are known to interact with membrane components [36–38]. Temperature-dependent changes in NAA levels as mentioned above might also indicate shifts in lipid metabolism to improve homeoviscous adaptation and oxygen supply. This substance also acts as the major donor of acetyl-groups for the myelinisation of neurons [39, 40].

Temperature had an effect on serotonin metabolism in both, *B. saida* and *G. morhua*, leading to higher HIAA/5-HT ratios at higher temperatures and indicating an increase of serotonin turnover. Similar results were observed in common carp by de Boeck et al., who suggested a Q_10_ effect, but no stress response, as a mechanism [41]. In contrast to our findings, Sebert et al. measured a reduced HIAA/5-HT ratio with rising temperature in the eel *Anguilla anguilla* [42], a finding which argues against a simple Q_10_ effect. However, serotonin is a modulator of respiration. Increased respiration through a risen O_2_ demand at higher temperature may also be reflected in the HIAA/5-HT ratio [43].

### Effect of CO_2_ on brain metabolites in *Boreogadus saida*

CO_2_ effects in *B. saida* were observed for the osmolytes MI and GABA. Interactive effects of temperature and CO_2_ were detected for glutamate. All of these observations can mainly be attributed to alterations at 8 °C, where high CO_2_ induced an increase in the concentrations of these three metabolites. These changes were quite small compared to temperature effects and were not significant in post hoc analysis. Additionally, CO_2_ caused a transient (non-significant) rise of glutamine and glutamate at 8 °C accompanied by a non-significant reduction of NAA. We suggest that these findings are symptomatic for shifts in neural energy metabolism at 8 °C which might be exacerbated by CO_2_. In mammals, NAA is coupled to the brain’s energy metabolism and may serve as an anaplerotic source of acetate and aspartate [44]. While acetate can enter the TCA cycle after activation with SH-CoA through acetyl-CoA synthase [45], the remaining aspartate can be transformed to oxaloacetic acid and subsequently acetylated to citrate to enter the TCA cycle as well [44]. Also, in a second pathway, aspartate becomes deaminated to oxaloacetic acid generating glutamate from α-ketoglutaric acid. Glutamate, glutamine and GABA concentrations in the brain are tightly coupled through the glutamate/GABA-glutamine cycle [35].

An increase of GABA and glutamate might also indicate greater GABAergic activity at 8 °C and high CO_2_, possibly reducing neuronal activity under limited energy conditions. After GABA release by inhibitory neurons, it is partly taken up by astrocytes and converted into succinate by GABA-transaminase and succinic semialdehyde dehydrogenase [46], simultaneously leading to the formation of glutamate from α-ketoglutaric acid. GABA is generated from glutamine via glutamate in neurons. This leads to an accumulation of ammonia, which is transported to astrocytes through the lactate-alanine shuttle [35]. Increased inhibitory activity of GABA is suggested to be responsible for the reduction of brain activity in response to hypercapnia in other vertebrates [47, 48]. A reduced neuronal energy demand may lead to an increase of lactate, as this metabolite serves as the favoured energy resource in neurons and is provided through glycolytic activity in astrocytes as part of the lactate-alanine shuttle [35]. An increase of lactate was not detected at 8 °C under high CO_2_ in *B. saida* indicating no surplus of energy substrates. Anaplerotic reactions to refuel the TCA cycle and subsequent oxidative decarboxylation may be crucial for energy supply in cold-adapted species, as these possess only low glycolytic capacity [40]. Strobel et al. found a CO_2_-induced reduction in succinate dehydrogenase activity in *Notothenia rossii* [21] and proposed an increased utilization of glutamate and aspartate in order to increase proton consuming decarboxylation processes, thereby supporting pH maintenance. We did not observe an increase in succinate levels at 8 °C in the high CO_2_ group of *B. saida*, indicating no inhibition of succinate dehydrogenase. However, glutamate und aspartate catabolism as well as GABA synthesis may still contribute to acid-base regulation.

MI increments at 8 °C in the high CO_2_ group remain enigmatic, but may serve as an alternative osmolyte to NAA in order to maintain osmolarity during NAA utilization. As CO_2_-induced behavioural alterations in *B. saida* were not affected by environmental temperature [9], we assume that the detected CO_2_-dependent metabolic changes at 8 °C are not the primary physiological cause of the CO_2_-induced behavioural alterations observed in this species. As an alternative explanation, CO_2_-dependent metabolic changes observed in *B. saida* at 8 °C may rather be symptomatic for temperature-induced energy limitation at 8 °C which is further exacerbated by environmental hypercapnia. The upper temperature limit of 8 °C detected by Kunz et al. in the animals used in this study supports this hypothesis [22]. Whether the observed generation of glutamate and GABA serve the formation of utilizable energy metabolites, the reduction of proton concentrations or indicate metabolic depression in order to reduce neuronal energy expenditures remains to be explored.

### Effect of CO_2_ on brain metabolites in *Gadus morhua*

In *G. morhua*, a CO_2_-induced increase of lactate was detected mainly at 8 °C with an additional significant interactive effect of temperature and CO_2_. Further interactions of CO_2_ and temperature-effects were observed for GABA and choline due to an increase at 8 °C in the high CO_2_ group. These changes occurred together with a non-significant increase of alanine, succinate, glutamate and glycine, while NAA remained largely unaffected. Although these findings seem somewhat similar to those in *B. saida* at 8 °C, we nevertheless suggest a different physiological causality involving increased GABAergic activity with unimpaired neuronal energy status. Simultaneous increases of succinate, alanine, glutamate and GABA might indicate an increased GABAergic activity as discussed above for *B. saida*; however, in *G. morhua*, the increase of GABA was detected with a simultaneous rise of lactate indicating an increase in the availability of this energy substrate. NAA as a marker of energy limitation displayed a non-significant trend to increase under high CO_2_, further indicating unimpaired energy status of the brain. Release of GABA and acetylcholine is coupled in some brain regions indicating an additional regulatory function of the latter [49–51] and might explain the significantly increased choline concentration. Interestingly, the high CO_2_ group of *G. morhua* at 8 °C not only differed from the high CO_2_ groups at other temperatures, but further displayed slightly reduced behavioural CO_2_-effects as discussed in Schmidt et al. [9]. Our results indicate that resilience against CO_2_-induced behavioural changes may be greatest in the middle of the thermal window; however, the mechanism behind this observation remains obscure.

### GABA-metabolism and its putative role for CO_2_-resilience

The question arises how an increase of GABAergic activity might protect the behaviour of fish, if altered GABA_A_-receptor activity is responsible for the behavioural impairments under OA scenarios [2]. At least in mammals, neurophysiological consequences of GABA release are quite flexible and an excitatory function of GABA through GABA_A_-receptor activity is already observed under normal conditions in the central nervous system [52]. Additionally, whether GABA is excitatory or inhibitory is dependent not only on the electrochemical gradient, but also on the timing and location of GABA release with respect to former excitatory postsynaptic potentials [52]. Thus, electrochemical acclimation or spatiotemporal adjustments of GABA release could sustain regular neuronal processing even under increased CO_2_.

One possible alternative explanation arises from the point of view that concentrations of tissue CO_2_ might be lower in the centre of an animal’s thermal window leading to increased resilience at optimum temperature. Studies allowing to address this hypothesis are scarce. Van Dijk et al. [53] found that CO_2_ partial pressure in white muscle of *Zoarces viviparus* rises at temperatures below the thermal optimum for somatic growth of this species [54]. Experimental conditions may also have interfered as the seawater *P*CO_2_ in the 8 °C high CO_2_ group was slightly lower than in the other high CO_2_ groups in *Gadus morhua* which should have led to a slightly lower tissue CO_2_ partial pressure in animals of this specific treatment group. Nonetheless, animals under optimum temperature conditions may be able to maintain the inhibitory function of GABA even under increased CO_2_. A simultaneous increase of GABAergic activity might lead to metabolic depression, as indicated by a simultaneous significant increase of lactate and GABA (Fig. 4a and c), and thus further reduction of tissue pCO_2_ which might also increase relative resilience as ambient CO_2_ rises. While the optimum temperature for growth in the animals used in this study is not known [22], Björnsson et al. found an optimum temperature of ~ 10 °C - 12 °C for Icelandic cod fed ad libitum at similar body weight [55, 56]. The optimum temperature of *G. morhua* depends on several factors including life stage, food supply and population [57]. Limited food supply reduces the optimum temperature for somatic growth [56]. Since animals were not fed ad libitum in this study, 8 °C may thus have been close enough to their thermal optimum. In further studies, blood pCO_2_ of cod under increased CO_2_ conditions should be investigated over a broad temperature range in order to test this hypothesis and to correlate the findings with their CO_2_ induced behavioural alterations.

## Conclusion

In conclusion, a temperature increase has similar physiological consequences in both, *Boreogadus saida* and *Gadus morhua* with strong temperature dependent alterations particularly among osmolytes, membrane components and metabolites involved in GABA metabolism. The long-term thermal limit of *B. saida* may have been reached at 8 °C, with energetic constraints exacerbated by an increase of environmental CO_2_. In *G. morhua*, no temperature-dependent alteration of the same metabolites has been observed indicating that the upper temperature limit had not been reached at 16 °C. Also, higher environmental CO_2_ did not elicit energetic limitation in *G. morhua*. Interestingly, we found a significant CO_2_ effect at 8 °C with a CO_2_-induced increase of lactate, GABA and choline which could be associated with increased behavioural resistance of *G. morhua* at this temperature. Our data indicate that *B. saida* is more strongly affected than *G. morhua* by the concomitant warming and CO_2_ increase expected to occur in the polar ocean until the end of the twenty-first century. In areas where the distribution of these species overlap, *G. morhua* might thus be more resilient to future OWA-related physiological challenges and might out-compete *B. saida* in the long term.

## Additional files

Additional file 1: Table S1. Group sizes and environmental CO_2_ partial pressure (*P*CO_2_, mean ± standard deviation after Kunz et al. [22]) of *Boreogadus saida* and Gadus morhua that were utilized for NMR and HPLC analysis. (XLSX 10 kb)

Additional file 2: Table S2. Protocol depicting the buffer composition throughout HPLC-analysis. Total measurement time per sample was 90 min. The first column shows the time for onset of the respective composition. (DOCX 11 kb)

Additional file 3: Table S3. List of treatment groups that violated normality-distribution for the respective components. (DOCX 13 kb)

Additional file 4: Figure S1. Boxplots depicting metabolite concentrations (relative to total creatine (tCr)) in the brain of *Boreogadus saida* at different temperatures and CO_2_ partial pressures. White shading indicates control, grey shading high CO_2_ partial pressure. Each box contains median, first and third quartile. Different letters indicate significant differences detected with Tukey HSD post hoc analysis (*p* < 0.05). Metabolites were sorted functionally in accordance with Table 1. (PDF 176 kb)

Additional file 5: Figure S2. Boxplots depicting metabolite concentrations (s.a.) in the brain of *Gadus morhua* at different temperatures and CO_2_ partial pressures. White shading indicates control, grey shading high CO_2_ partial pressure. Each box contains median, first and third quartile. Different letters indicate significant differences detected with Tukey HSD post hoc analysis (*p* < 0.05). Metabolites were sorted functionally in accordance with Table 1. (PDF 178 kb)

Additional file 6: Figure S3. Boxplots depicting the amount of 5-Hydroxyindoleacetic acid (HIAA) relative to Serotonin (5-HT) in the brain of *Boreogadus saida* (A) and *Gadus morhua* (B) quantified with HPLC. White shading indicates control, grey shading high CO_2_ partial pressure. Each box contains median, first and third quartile. Different letters indicate significant differences detected with Tukey HSD post hoc analysis (*p* < 0.05). (PDF 19 kb)

Additional file 7: Figure S4. Non-metric multidimensional scaling of metabolite/total creatine ratios in the brain of *Boreogadus saida*. Dots indicate individuals with colours representing the respective treatment temperature. Blue = 0 °C, green = 3 °C, red = 6 °C, yellow = 8 °C. (TIFF 937 kb)

Additional file 8: Figure S5. Non-metric multidimensional scaling of metabolite/total creatine ratios in the brain of *Gadus morhua*. Dots indicate individuals with colours representing the respective treatment temperature. Blue = 3 °C, green = 8 °C, red = 12 °C, yellow = 16 °C. (TIFF 937 kb)

Additional file 9: Raw data. (XLSX 30 kb)
