# Supplementary material for: Differences in neurochemical profiles of two gadid species under ocean warming and acidification
Source: Front Zool. 2017 Oct 30;14:49. doi: 10.1186/s12983-017-0238-5 (PMC5661927; doi:10.1186/s12983-017-0238-5)
Supplement: Supplementary file 2 — Protocol depicting the buffer composition throughout HPLC-analysis. Total measurement time per sample was 90 min. The first column shows the time for onset of the respective composition. (DOCX 11 kb) [file 12983_2017_238_MOESM2_ESM.docx]

| **Minute** | **Acetate-buffer/Acetonitrile [%]** | **Acetonitrile [%]** |
| --- | --- | --- |
| 0 | 88 | 12 |
| 60 | 80 | 20 |
| 62 | 60 | 40 |
| 64 | 40 | 60 |
| 66 | 20 | 80 |
| 68 | 0 | 100 |
| 72 | 20 | 80 |
| 74 | 40 | 60 |
| 76 | 60 | 40 |
| 78 | 80 | 20 |
| 80-90 | 88 | 12 |

Additional file 2: Table S2: Protocol depicting the buffer composition throughout HPLC-analysis. Total measurement time per sample was 90 minutes. The first column shows the time for onset of the respective composition.
