# Supplementary material for: Differences in neurochemical profiles of two gadid species under ocean warming and acidification
Source: Front Zool. 2017 Oct 30;14:49. doi: 10.1186/s12983-017-0238-5 (PMC5661927; doi:10.1186/s12983-017-0238-5)
Supplement: Supplementary file 3 — List of treatment groups that violated normality-distribution for the respective components. (DOCX 13 kb) [file 12983_2017_238_MOESM3_ESM.docx]

| Compound | *Boreogadus saida* | *Gadus morhua* |
| --- | --- | --- |
| Acetate | 3 °C Control CO_2_ |  |
| Acetyl-histidine |  |  |
| Alanine | 3 °C Control CO_2_  8 °C Control CO_2_ |  |
| Choline | 3 °C Control CO_2_  8 °C Control CO_2_ |  |
| Gamma-aminobutyric acid | 8 °C Control CO_2_ |  |
| Glutamine |  | 3 °C High CO_2_ |
| Glycerophosphocholine |  | 8 °C High CO_2_  16 °C High CO_2_ |
| Glutamate |  | 3 °C Control CO_2_  16 °C Control CO_2_ |
| Glycine | 3 °C High CO_2_ |  |
| Lactate |  |  |
| Myo-inositol |  |  |
| N-acetylaspartate |  |  |
| Posphocholine |  |  |
| Putrescine | 8 °C Control CO_2_ |  |
| Succinate |  | 3 °C Control CO_2_ |
| Taurine |  | 16 °C High CO_2_ |
| Trimethylamine *N*-oxide | 6 °C High CO_2_ | 8 °C High CO_2_ |

Additional file 3: Table S3: List of treatment groups that violated normality-distribution for the respective components.
