# Supplementary figures and images for: Differences in neurochemical profiles of two gadid species under ocean warming and acidification
Source: Front Zool. 2017 Oct 30;14:49. doi: 10.1186/s12983-017-0238-5 (PMC5661927; doi:10.1186/s12983-017-0238-5)

## Energy metabolism

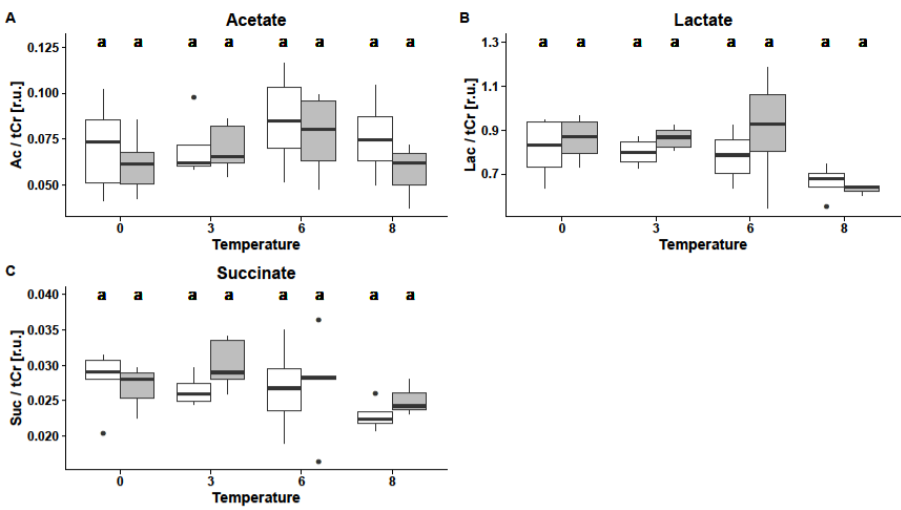

## GABA metabolism

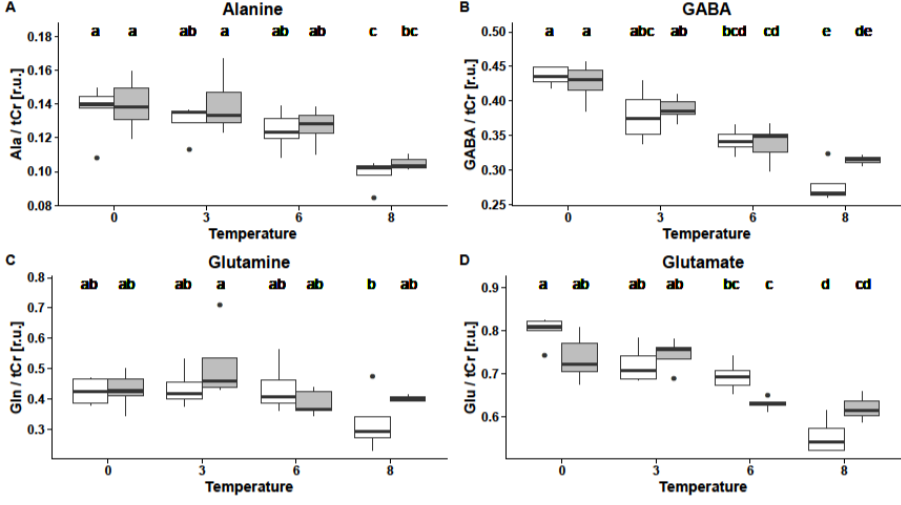

## Membrane components

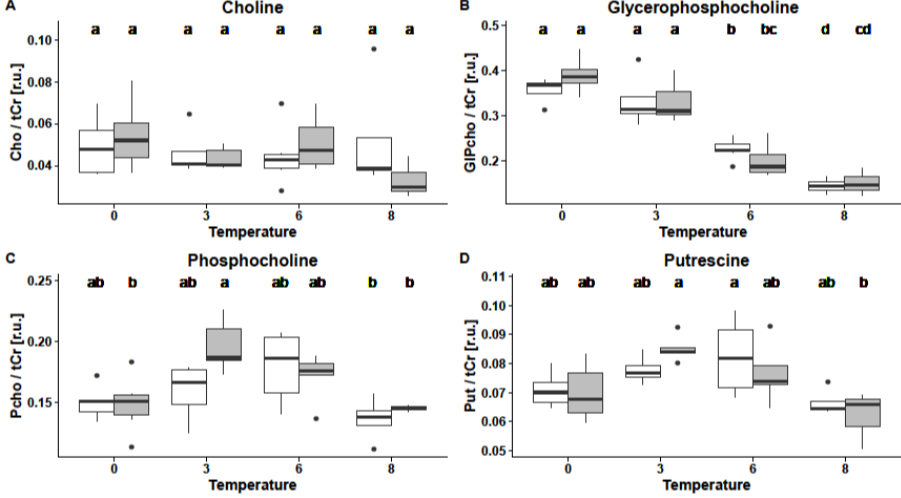

## Osmolytes

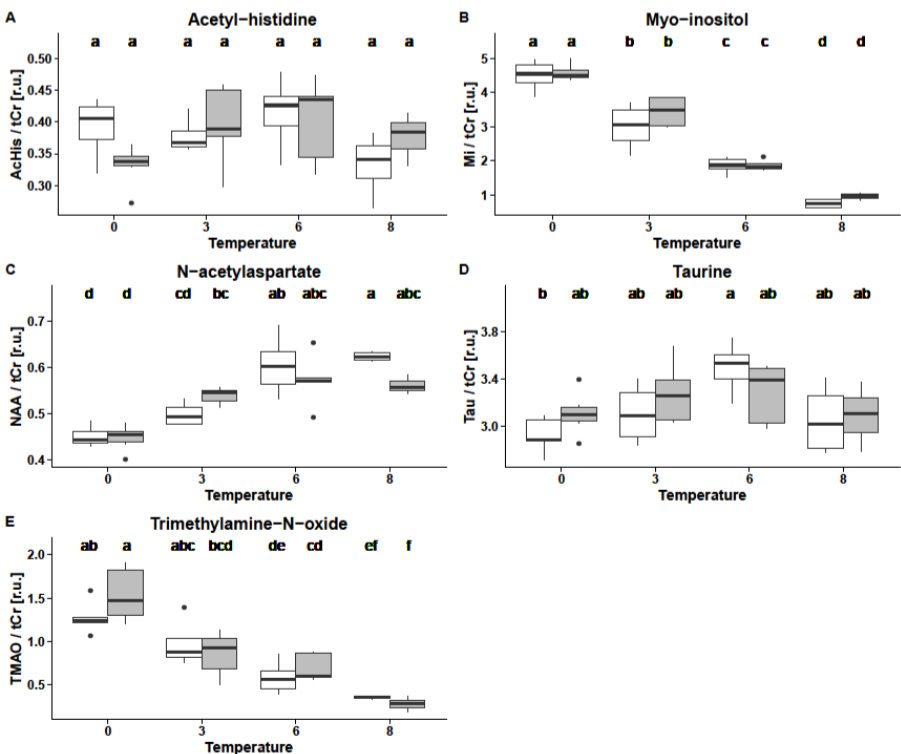

## Other

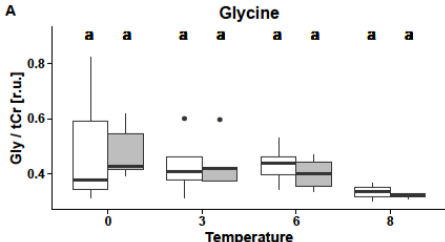

Supplement: Supplementary file 4 — Boxplots depicting metabolite concentrations (relative to total creatine (tCr)) in the brain of Boreogadus saida at different temperatures and CO2 partial pressures. White shading indicates control, grey shading high CO2 partial pressure. Each box contains median, first and third quartile. Different letters indicate significant differences detected with Tukey HSD post hoc analysis (p < 0.05). Metabolites were sorted functionally in accordance with Table 1. (PDF 176 kb) [file 12983_2017_238_MOESM4_ESM.pdf]

## Energy metabolism

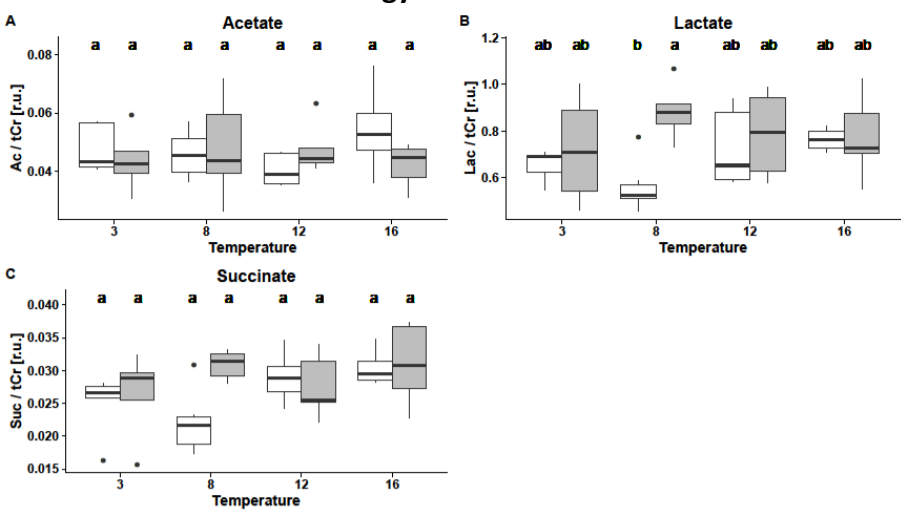

## GABA metabolism

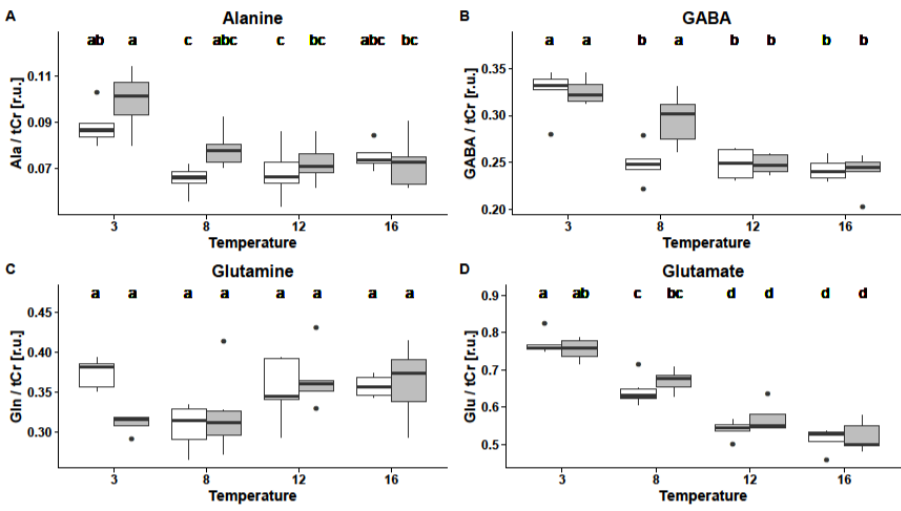

## Membrane components

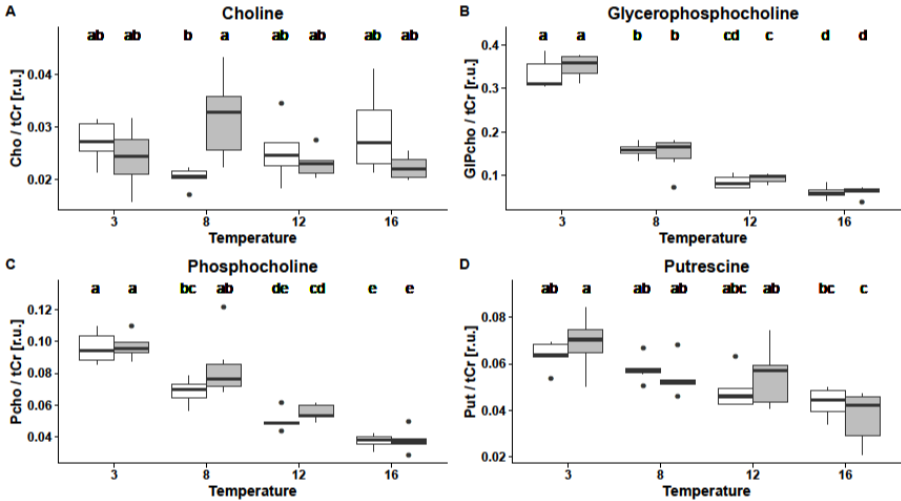

## Osmolytes

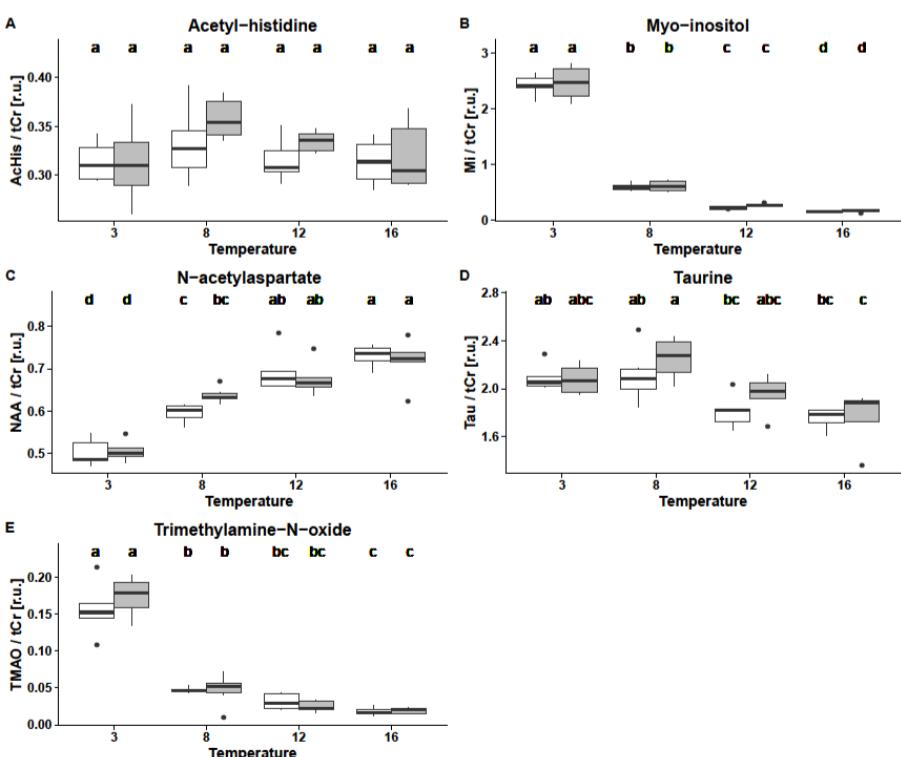

## Other

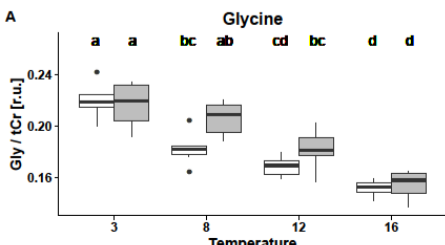

Supplement: Supplementary file 5 — Boxplots depicting metabolite concentrations (s.a.) in the brain of Gadus morhua at different temperatures and CO2 partial pressures. White shading indicates control, grey shading high CO2 partial pressure. Each box contains median, first and third quartile. Different letters indicate significant differences detected with Tukey HSD post hoc analysis (p < 0.05). Metabolites were sorted functionally in accordance with Table 1. (PDF 178 kb) [file 12983_2017_238_MOESM5_ESM.pdf]

**A**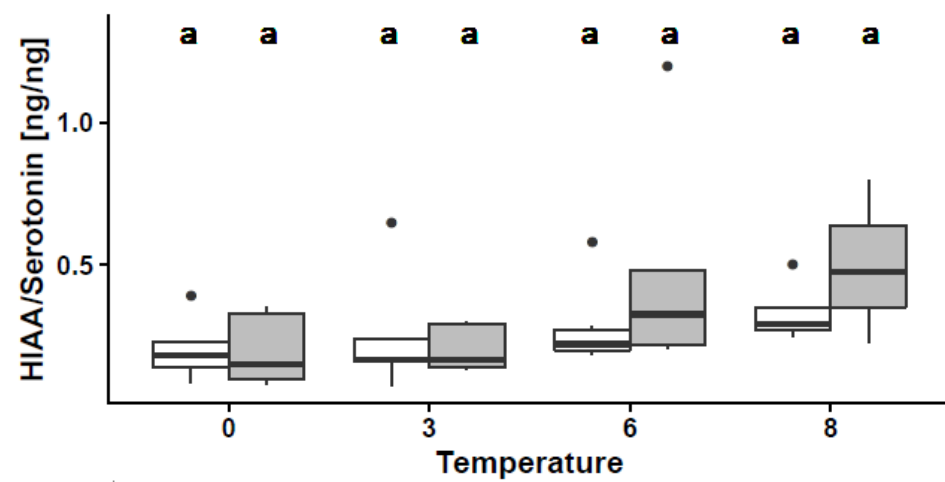**B**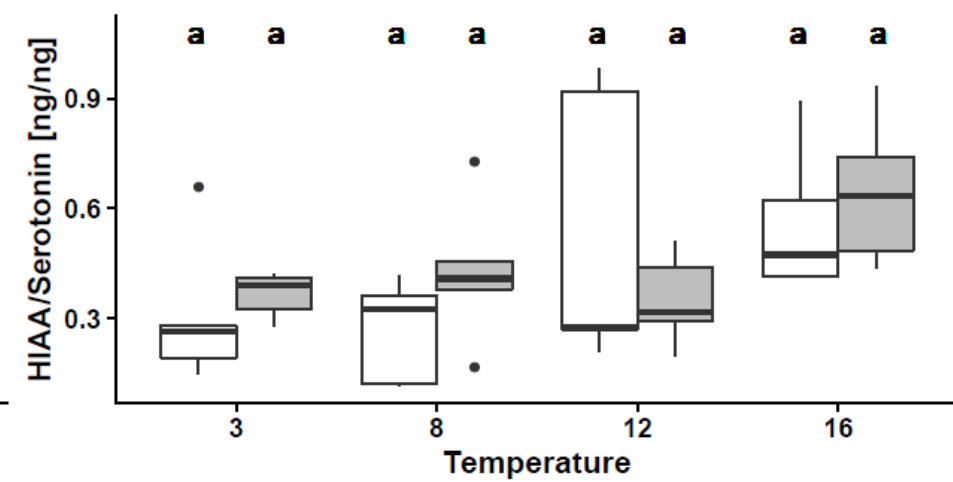

Supplement: Supplementary file 6 — Boxplots depicting the amount of 5-Hydroxyindoleacetic acid (HIAA) relative to Serotonin (5-HT) in the brain of Boreogadus saida (A) and Gadus morhua (B) quantified with HPLC. White shading indicates control, grey shading high CO2 partial pressure. Each box contains median, first and third quartile. Different letters indicate significant differences detected with Tukey HSD post hoc analysis (p < 0.05). (PDF 19 kb) [file 12983_2017_238_MOESM6_ESM.pdf]

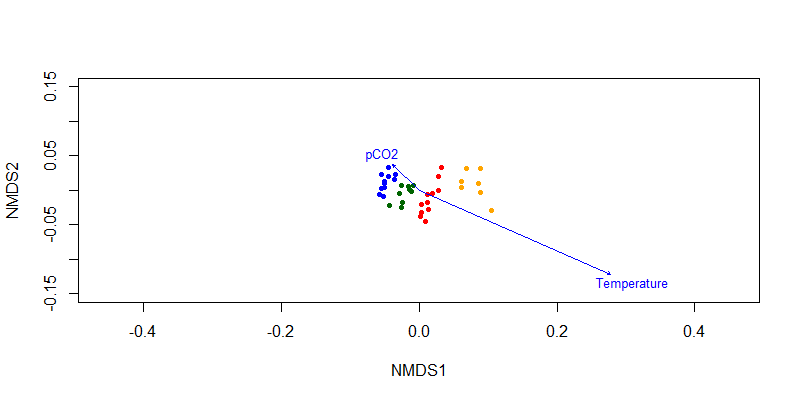

Supplement: Supplementary file 7 — Non-metric multidimensional scaling of metabolite/total creatine ratios in the brain of Boreogadus saida. Dots indicate individuals with colours representing the respective treatment temperature. Blue = 0 °C, green = 3 °C, red = 6 °C, yellow = 8 °C. (TIFF 937 kb) [file 12983_2017_238_MOESM7_ESM.tiff]

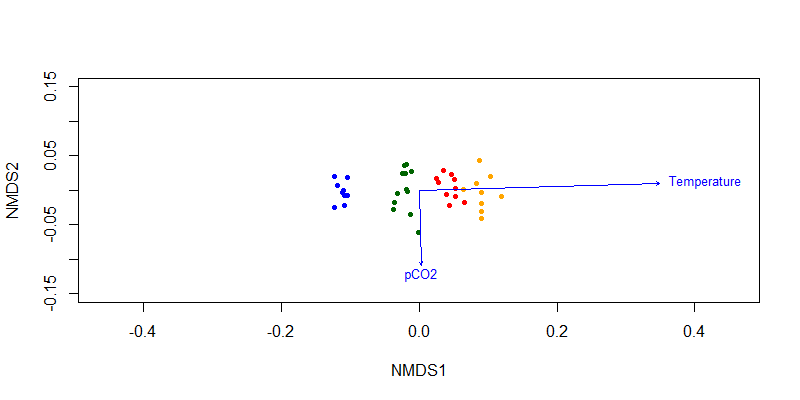

Supplement: Supplementary file 8 — Non-metric multidimensional scaling of metabolite/total creatine ratios in the brain of Gadus morhua. Dots indicate individuals with colours representing the respective treatment temperature. Blue = 3 °C, green = 8 °C, red = 12 °C, yellow = 16 °C. (TIFF 937 kb) [file 12983_2017_238_MOESM8_ESM.tiff]
